# The Combined Use of Correlative and Mechanistic Species Distribution Models Benefits Low Conservation Status Species

**DOI:** 10.1371/journal.pone.0139194

**Published:** 2015-10-01

**Authors:** Thibaud Rougier, Géraldine Lassalle, Hilaire Drouineau, Nicolas Dumoulin, Thierry Faure, Guillaume Deffuant, Eric Rochard, Patrick Lambert

**Affiliations:** 1 Irstea, EABX, Aquatic Ecosystems and Global Changes research unit, 50 avenue de Verdun, Gazinet Cestas, F-33612, Cestas, France; 2 Irstea, LISC, Complex Systems Engineering Laboratory, 9 avenue Blaise Pascal–CS 20085, 63178, Aubière, France; University of Porto, PORTUGAL

## Abstract

Species can respond to climate change by tracking appropriate environmental conditions in space, resulting in a range shift. Species Distribution Models (SDMs) can help forecast such range shift responses. For few species, both correlative and mechanistic SDMs were built, but allis shad (*Alosa alosa*), an endangered anadromous fish species, is one of them. The main purpose of this study was to provide a framework for joint analyses of correlative and mechanistic SDMs projections in order to strengthen conservation measures for species of conservation concern. Guidelines for joint representation and subsequent interpretation of models outputs were defined and applied. The present joint analysis was based on the novel mechanistic model GR3D (Global Repositioning Dynamics of Diadromous fish Distribution) which was parameterized on allis shad and then used to predict its future distribution along the European Atlantic coast under different climate change scenarios (RCP 4.5 and RCP 8.5). We then used a correlative SDM for this species to forecast its distribution across the same geographic area and under the same climate change scenarios. First, projections from correlative and mechanistic models provided congruent trends in probability of habitat suitability and population dynamics. This agreement was preferentially interpreted as referring to the species vulnerability to climate change. Climate change could not be accordingly listed as a major threat for allis shad. The congruence in predicted range limits between SDMs projections was the next point of interest. The difference, when noticed, required to deepen our understanding of the niche modelled by each approach. In this respect, the relative position of the northern range limit between the two methods strongly suggested here that a key biological process related to intraspecific variability was potentially lacking in the mechanistic SDM. Based on our knowledge, we hypothesized that local adaptations to cold temperatures deserved more attention in terms of modelling, but further in conservation planning as well.

## Introduction

Altered species distributions are typical responses of biodiversity to climate change [1, 2]. Contemporary latitudinal and elevational range shifts have been reported for many taxonomic groups in both terrestrial and aquatic ecosystems [3, 4]. Since the early 1990s, the future spatial distribution of species suitable habitats has been intensively projected using correlative Species Distribution Models (SDMs) [5]. However, there is increasing evidence that rapid (i.e., contemporary) evolutionary changes [6], dispersal [7, 8], spatial structures of the environment (e.g., habitat mosaics, connectivity between suitable patches) [9] and population dynamics [10] comprise factors that are just as important for determining future species ranges as the abiotic variables commonly considered in correlative SDMs. To improve predictions of species future distributions, there has been a recent development of hybrid models combining correlative SDMs and dispersal models e.g., [11, 12]. Mechanistic (i.e., process-based) SDMs that permit explicit incorporation of these complex range-limiting processes [13] can be integrated to further advance predictions. The effect of temperature on physiological and demographic processes, such as growth and survival, are regularly made explicit to test for a causal effect of temperature on species distributions [14]. However, mechanistic SDMs are more challenging to develop than correlative SDMs, especially at large scales, because they require more computationally intense processes, time, and data to be constructed, parameterized, and validated. For those reasons, joint analyses of SDM models for a given species remain rare but increasingly occur in the last few years [10, 15–19]. In this context, how results produced using different modelling methods, and sometimes in different studies and under the supervision of different researcher groups can be best exploited? The rationales needed for their joint interpretation, and particularly how results similarities and differences should be preferentially presented and interpreted, need clarifications.

Diadromous fishes have received attention from the scientific community regarding the simulation of their geographic distribution over time. Diadromous fishes migrate between fresh waters and the sea to complete their life cycle [20]. They have dramatically declined on a global scale during the last two centuries [21, 22]. Thirty-two percent of European diadromous fishes are currently ‘extinct’ or are ‘at risk of global extinction’ according to the International Union for the Conservation of Nature red list (www.iucnredlist.org). Given this poor outlook, the question of whether climate change could impact the effectiveness of diadromous fish conservation strategies has been raised. In response, correlative SDMs were first developed to quantify the future suitability of stocking river basins identified in national and European diadromous fish restoration plans [23, 24]. Diadromous fish were studied for decades because of their atypical life cycle associated with their economic, ecological and cultural importance [25]. This strong scientific basis enabled the recent development of a mechanistic SDM for diadromous fish named GR3D, i.e. ‘Global Repositioning Dynamics of Diadromous fish Distribution’ [26]. GR3D will help assess whether changing environmental conditions in river basins will allow existing populations to persist or new populations to become established using our current knowledge of the species population dynamics, the influence of temperature on key demographic parameters, and accounting for population source-sink dynamics.

The primary goal of this work was to provide a framework for the joint analysis of SDMs outputs to increase the robustness of model-derived conclusions, specifically towards resources managers involved in species conservation planning. We proposed guidelines on the representation of multiple models outputs, including graphical representations. We also defined guidelines for the interpretation of similarities and differences in models outputs both in terms of research activities and conservation planning. The present joint analysis was on the future diadromous fish distribution under climate change scenarios predicted by correlative and mechanistic SDMs. Using the allis shad (*Alosa alosa*) as a case study, we reported how GR3D was for the first time parameterized and calibrated following a sensitivity analysis and an optimization procedure. Two parameters were adjusted to match the data to be predicted using the Approximate Bayesian Computation (ABC) method. GR3D was finally implemented using climatic scenarios derived from the last Intergovernmental Panel for climate Change report [27] to predict species distributions until the year 2100. The allis shad correlative SDM and its projections were derived *de novo* following the procedure described in [28], but with updated global climate models and emission scenarios.

## Material and Methods

### General model presentation

#### Mechanistic model description

A full description of the GR3D model was provided by [26]. The Java code is available online following this URL http://trac.clermont.cemagref.fr/projets/SimAqualife/browser/GR3D_ECOMOD. The present work was conducted using this model. Here, we described the main model features and list all GR3D parameters in Table 1.

**Table 1:** GR3D parameter description with nominal values and ranges (minima and maxima) for the 11 parameters involved in the sensitivity analysis.

Parameter name	Description	Nominal value and range	References
Reproduction			
*repSeason*	Season of the reproduction	Spring	[29]
Δ*t*_*rec*_	Assumed age of juveniles produced by the reproduction (year)	0.33	[30]
*η*	Parameter to relate *S*_95,*j*_ and the surface of a spawning place (ind./km^2^)	2.4	[31]
* *θ*	Ratio between *S*_95,*j*_ and *S*_50,*j*_ in each spawning place	[1.8–2.2]	[31]
*a*	Fecundity of the species (eggs/ind.)	135 000	[32, 33]
* *surv*_*optRep*_	Optimal survival rate of an individual from eggs to the age Δ*t*_*rec*_	[1*10^−3^–5*10^−4^]	[31]
* ***TminRep*,** *ToptRep*, *TmaxRep*	**Water temperature (°C) regulating survival of an individual from eggs to the age Δ*t***_***rec***_	**[9–12]**, 20, 26	[32, 34]
* *λ*	Parameter to relate *c*_*j*_ and the surface of a spawning place	[3*10^−4^–5*10^−4^]	[31]
*σ*_*rep*_	Standard deviation of log-normal distribution of the recruitment	0.2	Expert opinions
*Sp*_*sp*_	Survival probability of spawners after reproduction	0.1	[29]
Downstream migration			
*downMigAge*	Age of individual when it runs toward the sea (year)	0.33	[30]
*downMigSeason*	Season of the run toward the sea	Summer	[32]
Growth			
*L*_*ini*_	Initial length of juveniles in estuary (cm)	2	[30]
*σ*_Δ*L*_	Standard deviation of log-normal distribution of the growth increment	0.2	Expert opinions
*L*_∞_	Asymptotic length of an individual (cm)	60	[35]
*TminGrow*, * *ToptGrow*, *TmaxGrow*	Water temperature (°C) regulating the growth	3, [15–19], 26	[35]
* ***k***_***optGrow***_	**Optimal growth coefficient (cm/season)**	**[0.2–0.5]**	[36, 37]
Survival			
* *Z*_*sea*_	Annual mortality coefficient at sea (year^-1^)	[0.2–0.6]	[31]
*H*_*riv*_	Annual mortality (different from natural) coefficient in river (year^-1^)	0	Expert opinions
* *TminSurvRiv*, *ToptSurvRiv*, *TmaxSurvRiv*	Water temperature (°C) regulating survival of individuals in river	[8–11], 20, 30	[32, 34]
*surv*_*optRiv*_	Optimal natural survival rate of individuals in river (year^-1^)	1	[32]
Maturation			
* *L*_*mat*_	Length at the first maturity (cm)	[36–44]	[32, 38]
Upstream migration			
*upMigAge*	Age of an individual when it runs toward the river (year)	-	[29]
*upMigSeason*	Season of the return of spawners in river for spawning	Spring	[29]
* *p*_*hom*_	Probability to do natal homing behavior	[0.6–0.9]	[39]
*α*_*const*_, *α*_*dist*_, *α*_*TL*_, *α*_*WA*_	Parameters of the logit function used to determine the weight of each accessible basin for dispersers/strays	-2.9, 19.7, 0, 0	Expert opinions
$\bar{D_{j-birthPlace}}$, *σ*_*j*−*birthPlace*_, $\bar{TL}$, *σ*_*TL*_, $\bar{WA}$, *σ*_*WA*_	Mean and standard deviation used for standard core values in the logit function	300, 978,-,-,-, -	Expert opinions
*w* ^*deathBasin*^	Weight of the death basin used to introduce a mortality of dispersers/strays	[0.2–0.6]	Expert opinions

* model parameters that were used in the global sensitivity analysis according to [26]. In bold were given the two most influential parameters according to the global sensitivity analysis. Complementary remarks regarding as to why nominal values and ranges were retained during model parameterization were given in [26].

**Fig 1:**
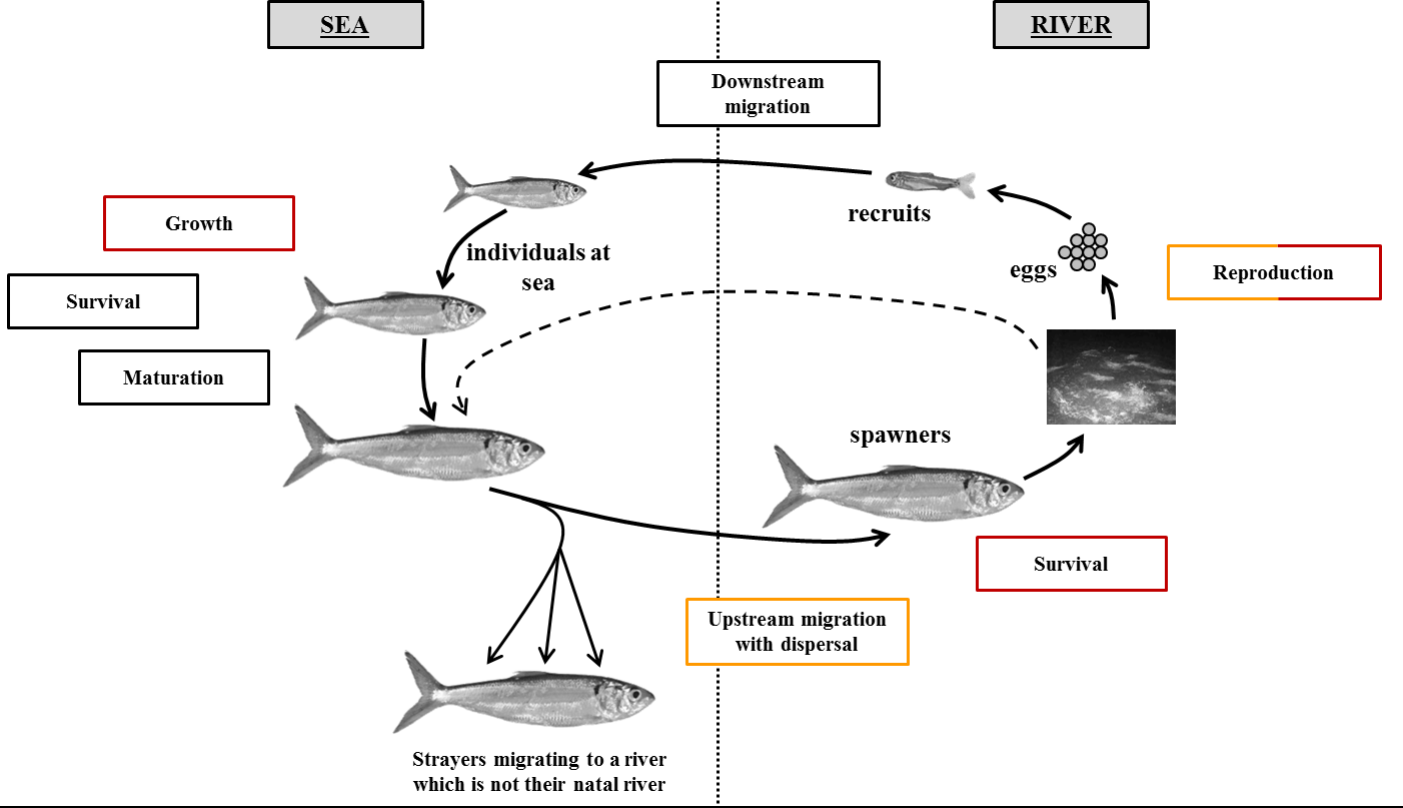
Conceptual diagram of the life cycle of anadromous species (adjusted to allis shad) represented in the GR3D model. Red boxes depicted the processes in GR3D that were influenced by temperature and orange boxes the ones that were linked to the surface area of the drainage basin. The figure was adapted from [26] for illustrative purpose only.

GR3D combines population dynamics, repositioning behavior through dispersal process and climatic requirements to assess local and global persistence, as well as potential changes to the distribution of diadromous fishes in response to climate change over large spatial scales. GR3D has been designed to provide a wide variety of modelling applications ranging from applied questions–where it can be parameterized for real landscapes and species as in the present work–to more theoretical studies of species dynamics under different environmental pressures. GR3D simulates a seasonal time step and has been designed to cover the entire life cycle of any diadromous fish species. The present GR3D application is centered on an anadromous species utilizing a specific computational order of life cycle events and processes as this type of diadromous species reproduces in fresh waters and grows at sea [20] (Fig 1 adapted from [26]).

In GR3D, reproduction of an anadromous species occurs annually during the reproductive season in each river basin when spawners are present. The number of recruits *R*_*j*_ (we assume that recruits are juveniles in estuaries) produced by *S*_*j*_ spawners in a river basin *j* is assumed to follow a Beverton and Holt stock-recruitment relationship (Eq 1). This equation is modified in two aspects. First, an Allee effect is included to take into account difficulties to establish a population with limited numbers of fish in new habitats [40, 41]. To do so, the Allee effect intensity (i.e., the number of spawners that effectively participate in reproduction) is modelled as a function of the river basin watershed area *wa*_*j*_ through the parameters *η* and *θ* (Table 1) with depensation intensity positively correlated to parameter *η* and negatively correlated to parameter *θ*. Secondly, a direct effect of water temperature and of watershed area on mortalities from eggs to recruits is considered on the parameters *α*_*j*_ and *β*_*j*_ of Eq 1 (see [26] for details):

$$R_{j}=\frac{\alpha_{j}S_{j}\frac{1}{1+e^{\left(-\text{ln}\left(19\right)\left(\left(S_{j}-\eta /\theta .wa_{j}\right)/\left(\eta .wa_{j}-\eta /\theta .wa_{j}\right)\right)\right)}}}{\beta_{j}+S_{j}\frac{1}{1+e^{\left(-\text{ln}\left(19\right)\left(\left(S_{j}-\eta /\theta .wa_{j}\right)/\left(\eta .wa_{j}-\eta /\theta .wa_{j}\right)\right)\right)}}}.$$ Eq 1

Growth of individuals is seasonal and modelled with a Von Bertalanfy growth function including an effect of water temperature *T* on the growth coefficient *κ* (Eq 2) through a dome shaped relationship [42] with an optimum growth coefficient *κ*_*optGrow*_ around *T*_*optGrow*_ and a null growth below *T*_min *Grow*_ and above *T*_max *Grow*_:

$$\kappa =\frac{\left(T-T_{\text{min}\;Grow}\right)\left(T-T_{\text{max}\;Grow}\right)}{\left(T-T_{\text{min}\;Grow}\right)\left(T-T_{\text{max}\;Grow}\right)-{\left(T-ToptGrow\right)}^{2}}.$$ Eq 2

Individual survival at sea depends on a fixed annual mortality coefficient *Z*_*sea*_ and survival in rivers depends on temperature, and is age-dependent.

While downstream migration is dependent on season and age, upstream migration is dependent on season and size. Upstream migration includes an original dispersal process which has been modelled as a three-stage process with emigration, transfer and settlement phases [43, 44]. During the emigration phase, individuals have a probability *p*_*hom*_ of adopting a homing behavior or 1−*p*_*hom*_ of adopting straying behavior. During the transfer phase, individuals that do not become strays migrate to their natal river. For strays, the probability to migrate in each river basin is assumed to be a function of its accessibility and its attractiveness. Accessibility is assumed to depend on dispersal distance and on the size of the individual. The basin attractiveness is assumed to be a function of its watershed area. Then, relatively to an individual, a weight is calculated for each river basin. Assuming that the individual may not find any basin and simply die during transfer, a virtual ‘death basin’ with a fixed weight in the environment is also introduced. Standardizing all the weights so that their sum equals 1, we provided a probability to choose each river basin (including the death basin) and we modelled the choice by a simple multinomial process. Then, during the settlement phase, individuals enter in the selected destination basin, survive if conditions are suitable and reproduce if they find mating requirements (see [26] for details).

#### Correlative model description

Full details of correlative SDM construction for European diadromous fishes are provided in [28]. In correlative SDMs, modelers search for the linear combination of environmental predictors that best reproduce the observed species distribution [5]. Then, these multiple correlations between realized species distributions and mostly abiotic predictors (e.g. climate) are used to assess habitat suitability under changing environmental conditions and with various purposes in ecology and conservation biology [45]. For the construction of diadromous fish SDMs, generalized additive models (GAMs; [46]) were used to relate presence-absence data of these species to a maximum of three biogeographic, geomorphologic, and climatic variables. The number of explanatory variables was limited to enhance the model accuracy and predictive power [5], the maximum of three variables being derived from [47]. All potential explanatory variables were carefully selected in accordance with major ecological theories, and the current understanding of diadromous fish biology and ecology [48]. These logistic regressions were fitted using a maximum likelihood method. Non-parametric smoothing functions with a degree of freedom restricted to two were used to test for linearly decreasing, increasing or dome-shaped response curves and to avoid model overfitting. The allis shad SDM presented in [28] had high performances in terms of reproducing past observed distributional patterns. It explained half of the deviance in the distribution data used during calibration and, the Kappa and AUC (Area Under the Curve) metrics categorized this model as ‘substantial’ to ‘good’, both during the calibration and validation phases. It was finally composed of three explanatory variables (i.e., longitude of the river basin outlet, summer air temperature at the basin outlet, and the surface area of the drainage basin). Allis shad thermal requirements in shape, range and optimum were in accordance with an experimental study on juvenile survival (Ph. Jatteau, pers. comm.).

### Biological and environmental data availability

#### Studied species

**Fig 2:**
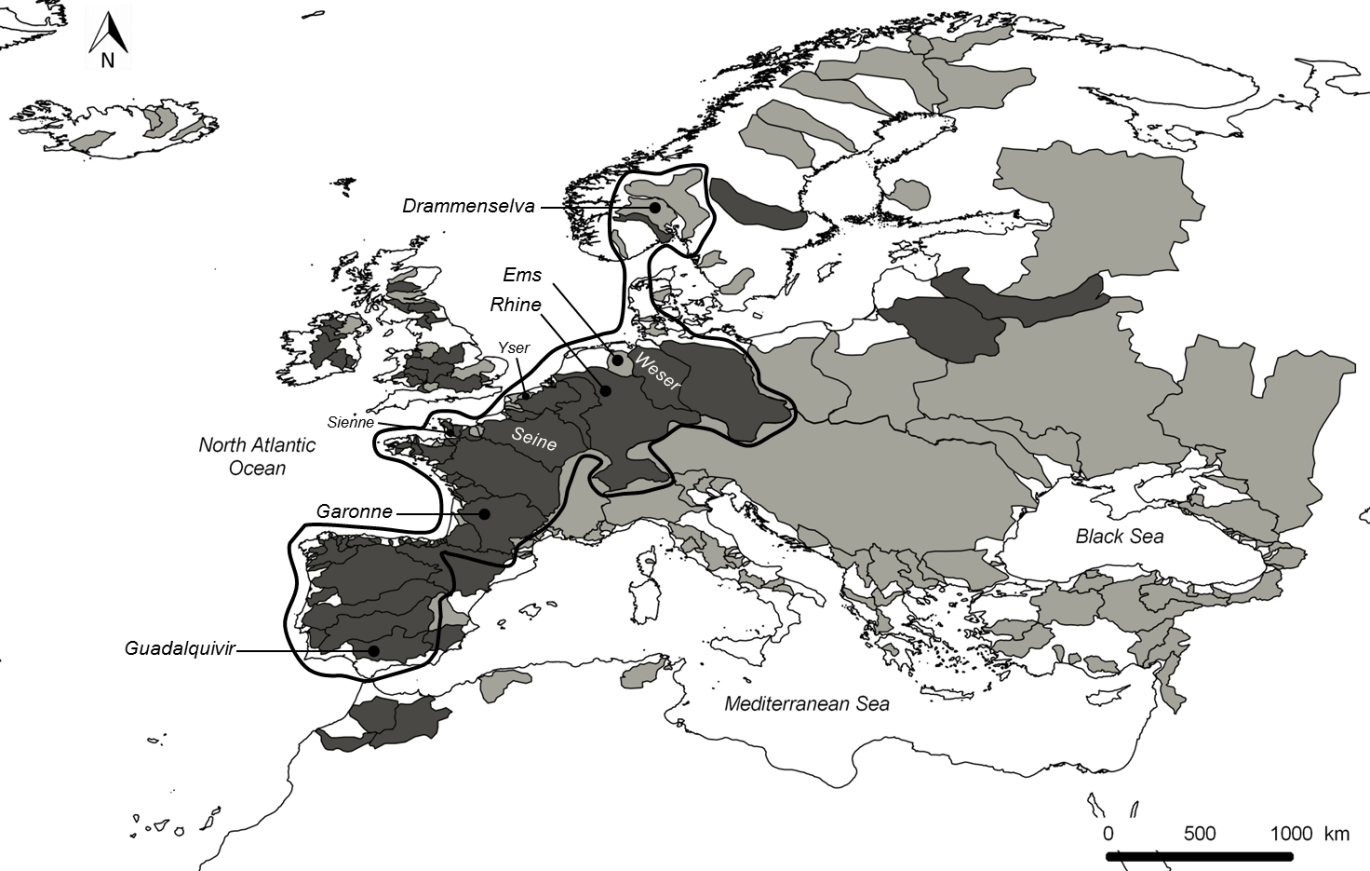
The geographical extent of the correlative and mechanistic modelling approaches with the allis shad historical distribution. Light grey and dark grey polygons corresponded to the 197 basins of EuroDiad 3.2 considered in the correlative SDM. Light grey and dark grey polygons represented also the allis shad former absences and presences around 1900, respectively. The area delineated by a solid black line denoted the 73 basins taken into account in the GR3D model application.

Allis shad (*Alosa alosa*) is an anadromous clupeid that spawns in the main stem of rivers. Fish migrate to sea during their first year where they grow and then return to fresh waters to spawn between 3 and 6 years old [49]. The species distribution (originally along the Atlantic coast from Norway to Morocco; Fig 2) has decreased considerably since the middle of the 20^th^ century, mainly because of overfishing, dam constructions, water quality degradation and deterioration of spawning habitats [22]. Currently, populations of allis shad exist along the northeastern Atlantic coast in some large rivers of France (i.e., Loire, Gironde-Garonne-Dordogne, and Adour) and Portugal (i.e., Minho and Lima) [38]. Despite the implementation of protective measures, this species appears to have been in serious decline for a number of years [22, 31]. Allis shad has lost nearly half of its populations in Europe since the mid-20^th^ century [50] and, for the Gironde population, long considered as the reference, a total fishing moratorium was implemented since 2008 due to a dramatic drop in landings [31]. Biology and ecology of allis shad have therefore received a great deal of attention in the last 30 years [30, 37, 51–56] and several studies also dealt with its population dynamics [31, 57]. This species is the focus of an ongoing stocking program (started in 2008) in the Rhine River (Germany) with juveniles coming from assisted reproduction of wild spawners from the Gironde-Garonne-Dordogne basin (France) [33]; http://www.lanuv.nrw.de/alosa-alosa/en/.

#### Biological data

Life history traits of allis shad required for the parameterization of the GR3D model were either obtained from the literature, or based upon expertise (see references mentioned in Table 1).

Data regarding the distribution of allis shad were obtained from the EuroDiad 3.2 database (Irstea, Cestas, France; available at http://www.diadfish.org/; see S1 Table). EuroDiad 3.2 describes the distribution of European diadromous fishes at three time steps (i.e., 1750–1850, 1851–1950 and 1951–2010). This database covers the Western Palearctic region including Europe, North Africa and the Middle East, with 197 river basins describing inland waters of those regions (Fig 2). The database records the presence or absence of European diadromous fishes in every basin. Allis shad were recorded ‘present’ in 79 basins in the first two time periods. Historical suitable basins were mainly in Western Europe, with a few being located in North Africa (Fig 2).

#### Physical environment and environmental data

The 197 river basins included in EuroDiad 3.2 were also described by key geomorphological attributes such as the coordinates at the outlet, the altitude of the source, the surface area of the drainage basin, and the length of the main watercourse (see S1 Table). Basins were also characterized by their climatic conditions averaged across the period 1901–1910 by seasons (i.e., winter: January, February and March; spring: April, May and June; summer: July, August and September; fall: October, November and December) and for the whole year. Historical near-surface atmospheric temperature at the outlet and precipitation across the whole basin were extracted from the recently up-dated CRU TS 3.22 database [58] which comprises monthly grids of observed climate for the period 1901–2013, covering the global land surface at 0.5 degree resolution (freely downloadable at http://www.cru.uea.ac.uk/cru/data/hrg/). Seasonal and annual mean air temperatures were converted into water temperatures by assuming a basic linear relationship between these two variables with water temperatures being 2°C lower than air temperatures.

For the mechanistic approach, all the basins in EuroDiad 3.2 could not be taken into account because of the time consuming computational processes required. Instead, 73 basins corresponding to the core distribution range of allis shad in northwestern Europe were retained to define the physical environment of GR3D. Retained basins were located along a latitudinal gradient between the Guadalquivir River in Spain (37°N) and the Drammenselva basin in Norway (59.80°N) along the North-Atlantic coast. Islands such as UK and Iceland were not included in this first mechanistic modelling attempt (Fig 2). The 197 basins described in EuroDiad 3.2 were considered in the correlative approach. Correlative SDMs require taking into account the entire biome to define proper species distribution edges (Fig 2).

### Calibration of models

#### Mechanistic model calibration

The GR3D calibration was based on simulating a stable allis shad distribution around 1900; consistent with the observed distribution of the species over the 1851–1950 period as described in the EuroDiad 3.2. database (hereafter: ‘historical distribution’). From this, three simulation summary statistics were designed.

The first two Summary Statistics (*SS*_1_ and *SS*_2_), were defined according to observed historical distribution patterns. In *SS*_*1*_, a predicted probability of each basin *j* to sustain a stable allis shad population *p*_*sust*1900,*j*_ was recorded. This probability relied on the amount of reproduction in the basin *j*, *N*_*rep*,*j*_, over the last 10 years of simulation (1891–1900):

$$p_{sust1900,j}=\frac{N_{rep,j}}{10}.$$ Eq 3

Then, based on the observed historical allis shad distribution, *SS*_1_ was computed as the following log-likelihood function:

$$SS_{1}=\sum_{j\in p}\text{log}\left(p_{sust1900,j}+\delta \right)+\sum_{j\in a}\text{log}\left(1-p_{sust1900,j}+\delta \right),$$ Eq 4

with *p* referring to all the basins where the species was historically recorded as present, *a* referring to all the basins where the species was historically recorded as absent and *δ* a constant fixed to 0.001 to avoid convergence problems.

In *SS*_*2*_, the latitude of the northernmost populated basin at the end of the simulation was recorded. To be considered as populated by allis shad, the basin should have a mean recruitment value over the last ten years of simulation above 50 juveniles. This criterion was based on the model exploration. Basins with a mean recruitment exceeding this value were not experiencing a crash of abundance at the beginning of the 20^th^ century. *SS*_*2*_ was later compared to the target value of 53.55 that corresponded to the latitude at the outlet of the northernmost basin where the species was historically recorded present in EuroDiad 3.2, (i.e., the Weser River in Germany).

The third summary statistic *SS*_3_ was defined according to an observed pattern since several studies showed that the mean age of allis shad spawners was five years-old [35, 59–61]. As such, the mean age of spawners ($\bar{SpAge_{j}}$) was recorded for each basin *j* considering only first mature spawners (i.e., not those which have previously spawned). *SS*_3_ was then computed as a sum of square of deviations from the target value of five years-old:

$$SS_{3}=\sum_{j}{\left(\bar{SpAge_{j}}-5\right)}^{2}.$$ Eq 5

From the definition of these three summary statistics, the calibration of GR3D was then run in two steps: (1) a global sensitivity analysis, and (2) an optimization procedure. The global sensitivity analysis was conducted to determine the two most influential model parameters on the three summary statistics [62]. The optimization procedure was run to determine a posterior distribution for these two influential parameters and to identify the influential parameter sets (couples) that best reproduced the historical distribution of allis shad in the GR3D physical environment [63]. Full details and outcomes of the calibration phase were given in S1 Appendix.

The GR3D global sensitivity analysis was performed on 11 uncertain parameters as identified by [26]. Maximum and minimum values for these parameters were determined with the main prerequisite to not exceed the 20% deviation from the best estimates (Table 1), commonly used in sensitivity analysis [64, 65]. A complete sampling was used meaning that all combinations of minimum and maximum values of the 11 uncertain parameters were defined and then run, resulting in 2^11^ simulations. To take into account the model stochasticity, each combination was also simulated 10 times, multiplying by 10 the total number of simulations. Sensitivity indices relative to a given parameter were calculated for each summary statistic. GR3D parameters not considered in the global sensitivity analysis were fixed to their nominal values given in Table 1. Uncertain parameters were finally classified by decreasing order of variations they caused on the three summary statistics. Two parameters were selected to be calibrated through the optimization step. These parameters had high values of sensitivity indices and a low interaction between them for a same summary statistic to make result interpretation more reliable and efficient.

The optimization procedure relied on a recent Approximate Bayesian Computation (ABC) algorithm specifically adapted to complex models [63]. The objective was to search for the sets of the two previous selected parameters within their min-max interval that maximized *SS*_*1*_ and that minimized *SS*_*3*_ with both a target value of 0, and that permitted *SS*_*2*_ to get closer to its target value of 53.55°N. We also obtained a posterior distribution for the two parameters. At this step, the nine other uncertain parameters were fixed to the central value of the min-max interval tested during the global sensitivity analysis.

During the whole calibration phase, each simulation started with the 73 basins of the GR3D physical environment characterized by their mean seasonal water temperatures for the period 1901–1910 and their surface area. They were all populated by 500 000 juveniles at the first time step which was in summer. Simulations lasted 100 years with constant climatic (i.e., temperature) conditions mimicking the 1901–1910 period.

#### Correlative model calibration and validation

To enhance mechanistic and correlative model joint analysis, the correlative model was calibrated and validated *de novo* with the same climatic data as used for the mechanistic model, and following the procedure described in [28]. The surface area of the drainage basin was also log-transformed to account for extreme values corresponding to large Middle Eastern basins (e.g., the Ural and Volga rivers).

Correlative model calibration and validation aimed at reproducing the historical species distribution. In this case, all linear combinations of one, two or three potential explanatory variables were compared against the Akaike Information Criterion AIC; [66]. The combination with the lowest AIC value was retained and its ability to reproduce the historical allis shad distribution was evaluated with the Kappa coefficient which measures the proportion of species entries correctly classified as presences (sensitivity) or absences (specificity) after the probability of chance agreement has been removed [67], the Area Under the Curve (AUC) that relates sensitivity and false positive proportion (1-specificity) over a wide and continuous range of threshold levels, which makes it a threshold-independent measure [68], and the percentage of deviance explained by the model calculated as follows:

$$ExpDev=\frac{Null\;model\;deviance-Final\;model\;deviance}{Null\;model\;deviance}\times 100$$ Eq 6

The Null model only contained the intercepts. For Kappa calculation, the probabilities of a basin to be suitable to allis shad derived from the model needed to be transformed into presences-absences using a threshold comprised between 0 and 1. Here, the threshold maximizing the Kappa value was the one applied. Historical allis shad distribution and potential environmental explanatory variables were extracted from the EuroDiad 3.2 database. Among the 197 river basins describing inland waters of the Western Palearctic region in this database, the species was recorded present in 79 basins and absent in 118 (Fig 2; see S1 Table). Seventy-five percent of species entries from the database were used in model calibration and the remaining part in model validation. Model validation consisted of evaluating model predictive performances on data not utilized during model calibration using the Kappa and AUC metrics.

### Projection of allis shad dynamics and habitat suitability for 2100

Future climatic conditions were obtained from dynamical downscaling of Global Climate Model (GCM) projections performed in the scope of the fifth Intergovernmental Panel for Climate Change (IPCC) assessment report (AR5) [27]. EURO-CORDEX is the European branch of the international CORDEX (Coordinated Regional Climate Downscaling Experiment) initiative, which is a program that aims at producing improved regional climate change projections for all land regions world-wide [69] (http://www.euro-cordex.net/). The outputs (2014–2100) of monthly near-surface air temperature and precipitations from the RCA-4 regional climate model was used in this study, with the following available ‘Representative Concentration Pathways’ scenarios: the medium RCP 4.5 and the high RCP 8.5. Downscaled projections were limited to one GCM, the CNRM-CM5. Data at 0.5 degree resolution can be freely downloaded at http://pcmdi9.llnl.gov/esgf-web-fe/.

For GR3D, a simulation lasting 300 years (i.e., 1200 seasonal time steps) was run. During the first 100 years of simulation, water temperatures were fixed to the 1901–1910 mean (i.e., initialization of a stable allis shad distribution around 1900).Then, temperatures evolved according to the CRU TS 3.22 database (i.e., reproduction of temperature evolution of the 1901–2013 period) and to the selected RCP scenarios (i.e., simulation of temperature evolution for the 2014–2100 period). This 300 years simulation was run 100 times with 100 couples of the two calibrated parameters; their values being randomly sampled in their posterior distributions. The predicted probability of a basin to sustain a stable allis shad population *p*_*sust*2100,*j*_ over the 2070–2100 period was recorded in each basin *j*. This probability relied on the amount of reproduction *N*_*repFinal*,*j*_ over the last 30 years of simulation:

$$p_{sust2100,j}=\frac{N_{repFinal,j}}{30}$$ Eq 7

For the correlative SDM, potential future basin suitability was projected by changing the climate as predicted by RCA-4 under the two available RCP scenarios. Temperatures were averaged over thirty years from 2070 to 2100 to smooth inter-annual variability and were entered in the model. These simulations provided a probability *p*_*suit*2100,*j*_ for each basin *j* to be suitable for allis shad at the end of the 21^th^ century.

### Joint analyses of SDM results: Guidelines

One objective of this paper was to enhance joint analyses of correlative and mechanistic SDMs results when the two exist for a given species. As a first guideline regarding results representation, (1) a focus should be made on the geographic entities common to the two modelling approaches, i.e. here the 73 basins that constituted the GR3D physical environment. (2) The same evaluation metrics need to be calculated during both model constructions. The commonly-used Kappa coefficient, percentages of well-predicted presences/absences and AUC metric were calculated for both the correlative SDM and the GR3D model. They were then used to evaluate the models ability to reproduce the historical species distribution. In GR3D, a basin was considered populated in 1901–1910 when the mean recruitment value over the final ten year period of the first 100 years simulation was >50 juveniles, as described for the *SS*_*2*_ summary statistics. This binomial (populated/not populated) variable was used during the GR3D Kappa calculation and *p*_*sust1900*_ was used for the GR3D AUC calculation. As SDMs are often developed to be used for more than one purpose, e.g. for diverse applications from conservation biology to invasion ecology [70], it is preferable to provide raw data (i.e., probabilities) and to allow users to apply thresholds to produce binary or categorical outputs, if necessary. (3) Simulations of the future species distribution have to be analyzed together using probabilities: probabilities for each basin *j* to be suitable in the correlative SDM (i.e., *p*_*suitFinal*,*j*_) and probabilities for each basin *j* to sustain stable populations in the mechanistic SDM (i.e., *p*_*sustFinal*,*j*_). Probabilities were also converted here into five classes (i.e., 0: null,] 0–0.25]: low,] 0.25–0.53]: moderate,] 0.53–0.75]: high and] 0.75–1]: very high). The limit 0.53 was retained between the moderate and high classes as this value represented the threshold maximizing the Kappa index in the correlative SDM. (4) We assumed that the representation and interpretation of probabilities on maps when the model units were not of fixed geographical area such as pixels could be partly biased. For example, when representing surface area of the drainage basins on maps, the importance of results in large entities could be over-interpreted. Therefore, heat maps and bivariate plots were here proposed as complementary representations.

Regarding joint model interpretation, guidelines were centered on how similarities and differences in models outputs can be interpreted in terms of research activities and conservation planning (Table 2). Two basic patterns were addressed: (1) The overall trend in habitat suitability and population dynamics in response to future climate change. Do the two approaches gave the same broad picture? (2) The distribution range limits predicted under past and future climate conditions. Do the same geographical extent covered by the two approaches? The first will contribute to the improvement of our knowledge on species climate change vulnerability and conservation status [71, 72] while the second will help in enhancing our mechanistic understanding of species distribution limits [73] and, by extension, the reliability of conservation measures.

**Table 2:** Guidelines for interpreting similarities and differences in SDMs outputs in terms of research activities and biological conservation. In bold were given the categories to which the present study was finally assigned. Blank cells signified that preferentially no conservation planning recommendations should be drawn. SDM_C_ and SDM_M_ were abbreviations corresponding to the correlative and mechanistic species distribution models, respectively.

	Trend between predicted past distribution and predicted future distribution (probabilities: increasing, decreasing, stable)			Past and future predicted range limits		
	Robust forecasts of climate change response	Divergent forecasts of climate change response	Present study	Congruent range limits	Wider or narrower range limits	Present study
Research activities	**Assessment of the vulnerability to climate change was improved**	A need for comparing the response curve to the climate component (e.g. temperature in SDM_C_) with the functional relationships linked to climate (SDM_M_)	**Low concern for allis shad, at least when considering temperature**	Mechanisms determining the species range limits in the SDM_C_ were most likely well-known as explicitly integrated in the SDM_M_	**A need for clarifying the niche most likely modelled by each SDM as the interpretation will be related [13, 73]**	**SDM**_**C**_ **more closely related to the realized niche and SDM**_**M**_ **to the fundamental niche. Population local adaptation is suspected to be required in the SDM**_**M**_ **and should be tested before field or experimental validation**
Conservation planning	**Revision of the conservation status in light with climate change effect [71, 72]–Categorization of threats**		**Climate change alone (i.e. temperature) should not be listed as a major threat**	Assessment of the adequacy of key conservation measures to these mechanistic insights		

## Results

Compared to the first modelling attempt of [28], the use of the latest version of the CRU TS database to construct the allis shad correlative SDM did not change the explanatory variables retained in the model nor their response curves. Regarding climatic variables, summer air temperature was also selected with an optimal value around 16°C. The sensitivity analysis performed on GR3D identified two of the most influential model parameters which were associated with two distinct processes (i.e., survival and growth; Table 1), and which were directly linked or influenced by climate. *T*_*minRep*_ (the temperature below which the eggs and larvae survival was null) was an important factor in the capacity of GR3D to maintain populations at northern latitudes. *k*_*optGrow*_ (or the optimal growth coefficient) was crucial in mimicking the observed pattern of first reproduction at 5 years-old (see S1 Appendix). Posterior distributions of the two parameters were both unimodal curves with modal values consistent with the knowledge regarding allis shad biology and ecology (see S1 Appendix). The generation of these posterior distributions required 106 000 simulations and a computing time of *c*. 62 hours.

Models predictive performances evaluated on their ability to reproduce the historical species distribution in terms of presences/absences were moderate for the mechanistic SDM and high for the correlative SDM (Kappa values of 0.46 and 0.75, and AUC values of 0.75 and 0.95 respectively; Table 3). Both models correctly reproduced the observed presences of allis shad around 1900 (63% and 94% respectively; Table 3). However, only the correlative model accurately reproduced the known absences of the species (83% of absences well predicted versus 16% for the mechanistic model; Table 3). This percentage of absences well-predicted by the correlative model was nonetheless reduced by more than three (i.e., 25%) when considering only absences reported in the 73 basins that constituted the GR3D physical environment. GR3D delineated a homogeneous historical distribution along the northern Atlantic coast with few basins that did not exhibit a stable population in between.

**Table 3:** Predictive performances of the correlative and mechanistic SDMs. Values in brackets corresponded to percentages when only basins from the GR3D physical environment were considered (73 over 197).

	Correlative SDM (SDM_C_)	Mechanistic SDM (GR3D / SDM_M_)
Number of basins	197	73
Explained deviance	54.0	-
Kappa statistics	0.75	0.45
AUC statistics	0.95	0.75
% of presences well-predicted	94.0 [98.0]	63.0
% of absences well-predicted	83.0 [25.0]	15.9

**Fig 3:**
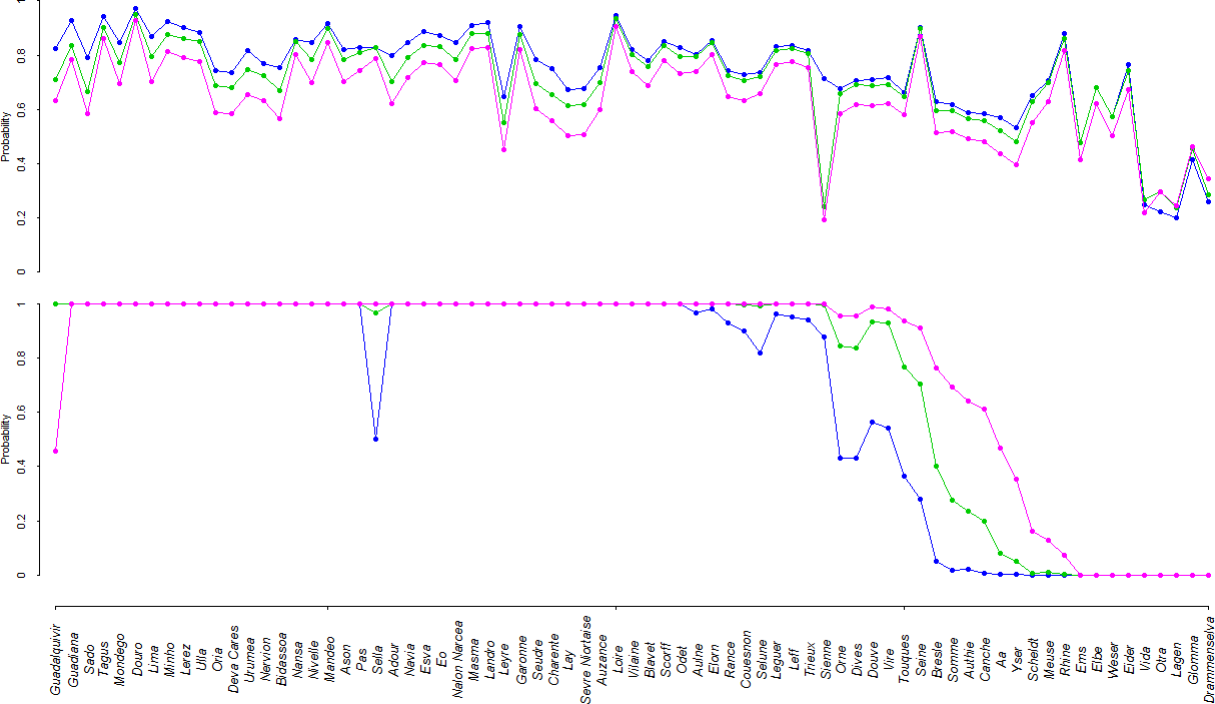
Species distribution models outputs along the latitudinal gradient from southern Spain to southern Scandinavia. (a) The upper panel represented outputs of the correlative SDM and (b) the lower panel the outputs of the mechanistic SMD. Blue, green and pink circle symbols represented probability outputs for 1901–1910, for 2070–2100 assuming the RCP 4.5 scenario and for 2070–2100 assuming the RCP 8.5 scenario respectively. For the correlative model, probabilities corresponded to the probability for a basin to be suitable at the given time period while for the mechanistic SDM, it represented the probability for a basin to sustain a stable population.

**Fig 4:**
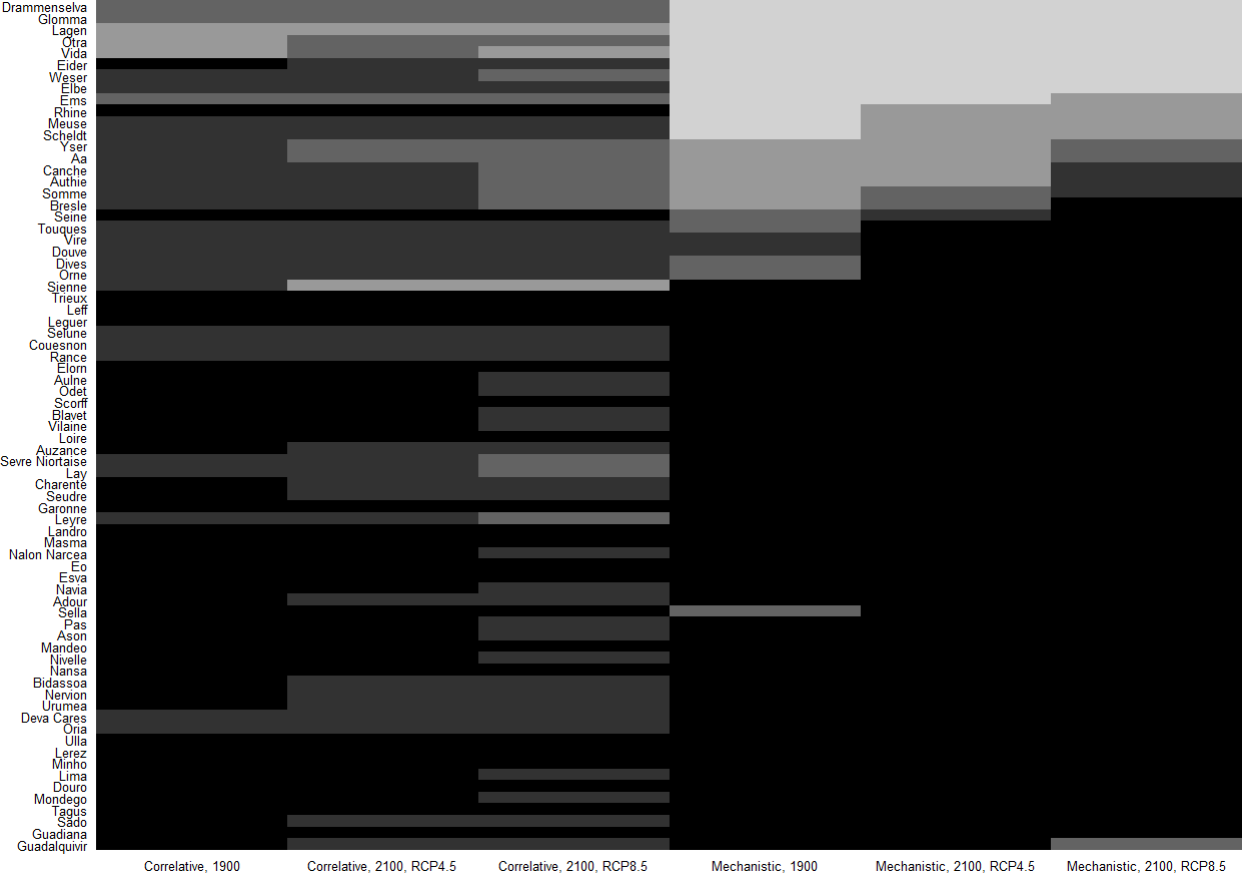
Heat map representing the probability classes for the 73 basins at the species historical core distribution range for the two times steps, i.e. 1901–1910 and 2070–2100, and for the two modelling approaches, i.e. the correlative and mechanistic SDMs, and the two climate change scenarios, i.e. RCP 4.5 and 8.5. Five classes 0,] 0–0.25],] 0.25–0.53],] 0.53–0.75], and]0.75–1] were represented by a continuous grey gradient with black used for the highest probability class] 0.75–1]. Basins were ordered along a latitudinal gradient (i.e., latitude at the basin outlet) from South (i.e., Guadalquivir) to North (i.e., Drammenselva).

Regarding trends in predicted probabilities (Table 2), one strong and common feature of SDMs predictions was probabilities of basins to be suitable for allis shad (*p*_*suit*2100,*j*_ predicted by the correlative model) and to sustain stable population (*p*_*sust*2100,*j*_ predicted by the mechanistic model) remaining high around 2100 under both RCP scenarios (Fig 3A and 3B). The average probability of a basin to be suitable around 2100 (i.e., $\bar{p_{suit\;2100}}$) was 0.71 and 0.64 under RCP 4.5 and 8.5 respectively. The average probability of a basin to sustain a stable population (i.e., $\bar{p_{sust\;2100}}$) was 0.76 and 0.80 under RCP 4.5 and 8.5 respectively (Fig 3A and 3B). In addition, $\bar{p_{suit\;2100}}$ and $\bar{p_{sust\;2100}}$ exhibited few changes compared to the 1901–1910 period as $\bar{p_{suit\;1900}}$ and $\bar{p_{sust\;1900}}$ equaled 0.74 and 0.69 respectively (Fig 3A and 3B). More specifically, for basins at the core of the species distribution, the GR3D model showed probabilities remaining stable and close to 100% under both RCP scenarios (Fig 3B and Fig 4). Up to the Sienne basin in France, reproduction was predicted to occur every year over the 30-year period for the two RCP scenarios (Fig 3B and Fig 4).

Regarding the past and future predicted range limits (Table 2), the correlative SDM captured the full observed distribution range, whereas GR3D showed difficulties in correctly reproducing the northern limit of the historical distribution (Figs 3A, 3B and 4), leading to the lower percentage of well-predicted presences (Table 3). For basins at latitudes higher than the Seine estuary, GR3D predicted in 1901–1910 an average recruitment over the period systematically below the ‘50-juveniles’ threshold and a probability of sustaining a stable population below 0.20 (Fig 3B). Nevertheless, false presences or absences in the correlative SDM also concerned northernmost basins. Under climate change, in a fringe composed of northern French, Belgian, Dutch, and German basins (i.e., between the Yser basin and the Ems basin), probabilities of basins to sustain stable reproductions were passing from almost zero in 1901–1910 to intermediate values for the end of the 21^st^ century (Figs 3B and 4). This highlighted an additional important result emerging from the mechanistic SDM simulations which was that allis shad dispersal capacities (as they are explicitly represented in the GR3D model) enabled the species to colonize new suitable watersheds farther north during this 200-year period of simulation. Secondly, at the southern edge of the distribution, a marked decrease in probability was predicted under the RCP 8.5 scenario for the Guadalquivir basin (Figs 3B and 4), probability being divided by a factor of two.

## Discussion

In our application case, to further understand the relative roles of different parameters in the mechanistic model outputs and to avoid misinterpretation of simulation results, we used a global sensitivity analysis to identify two of the most sensitive model parameters that we then calibrated using a recent ABC algorithm adapted to complex stochastic models [63]. As far as we know, this is one of the first attempts to calibrate such a complex mechanistic SDM using observed data (see [74] for a non-exhaustive list of fitted process-based models). Despite of the recent works in the field of complex model calibration e.g., [63, 75, 76], this process remains computationally heavy, but affordable in regards to the benefit of this paradigm, i.e. identify a more realistic model structure and parameter sets.

To date, there have been few direct combined uses of correlative and mechanistic SDMs for the same species, but the number of species with multiple opportunities of species distribution modelling is increasing. In the present study, we argued that combining both modelling approaches may improve the use of SDMs in conservation planning and management under climate change [77]. We proposed a conceptual framework for the SDMs outputs representation and interpretation (following two main patterns summarized in Table 2), with particular attention on insights that can be incorporated into conservation planning. From the present joint modelling attempt, allis shad exhibited robust and optimistic responses to future climate change under both moderate and pessimistic climate change scenarios: basins displaying suitable environmental conditions and basins with a high probability to support self-sustaining populations (while other human-induced pressures were considered to their pristine level) were predicted to remain quite stable according to the correlative and mechanistic SDMs, respectively. In the latter approach, the number of basins with a moderate to high probability of hosting a self-sustaining population was also increasing during the 21^st^ century, with gains at the northern species range. Indeed, the mechanistic SDM was parameterized with a homing rate of 0.75 (i.e., straying rate of 0.25) allowing shads to explore the environment and to colonize basins farther north than the 1900-calibrated northern edge. The homing rate used in the model was the most probable estimate based on current knowledge and was in accordance with a recent study on natal origin determination through otolith microchemistry analysis [78]. Consequently, these modelling results strongly suggest that allis shad may be able to cope successfully with ongoing climate change that should not be as such perceived as a major threat to the species long-term persistence. This result is consistent with other statistical and modelling studies highlighting that no significant environmental effect in allis shad abundance time-series has been identified in two major French basins still holding a self-sustaining allis shad population [31, 79]. Moreover, for a sympatric species, *A*. *fallax*, future climate change is likely to be beneficial to populations [28], and is further predicted to increase survival and population persistence in U.K. rivers [80]. For another related species *A*. *sapidissima*, 21^st^ century climate change is of great concern, as it is strongly suspected to favor population expansion of the species in its introduced range along the American Pacific coast up to Alaska and beyond [81]. Secondly, jointly interpreting results in terms of predicted range limits raised, in the first place, questions regarding the niche modelled by these two methods [82–84]. It is not possible to make a definitive statement about exactly what niche is being model by correlative SDMs, especially when no biotic variables were tested or included in the model. Nonetheless, correlative models statistically relate environmental variables directly to species occurrences or abundance. Thus, it implicitly incorporates any biotic interactions that are dependent on the abiotic variables considered [73]. In the present model, temperature is highly influencing the fish metabolism but also e.g. the dynamics of their zooplanktonic prey. The surface area of the drainage basin was associated to the species-area theory implying that species richness increases as a power function of the surface area (see [28] for variables ecological interpretation). In this respect, the present correlative SDM was considered as more closely related to the realized niche. In mechanistic SDMs, organisms are described as a set of behavioral, morphological and physiological traits. Mechanistic SDMs explicitly incorporate population dynamics with special focus on processes that limit species distributions. This represents a mechanistic depiction of a species fundamental niche which can then be used to infer distribution limits [13]. In the present work, the fundamental niche predicted by the mechanistic SDM was smaller than the potential observed/realized niche suggesting that within-species variability was very likely at play at the northern range edge. Indeed, the limit position in GR3D simulations was demonstrated to be particularly sensitive to the temperature parameter *T*_*minRep*_ linked to survival of eggs and larvae. We hypothesized that adaptation of local populations had occurred across generations favoring the survival of shad young stages to lower temperatures as it has been shown that local adaptation is frequent in salmonid populations [85]. Nonetheless, integrating all the processes involved in a species distribution in mechanistic SDMs still remain challenging as it increases model complexity and makes it more difficult to calibrate, and to extrapolate and analyze model outcomes [86, 87].

More specifically, when thinking about shad conservation, the present results bring new insights on the relevance of assisted colonization [88] and stocking programs. Over the past decade, a debate has evolved in the scientific community over the costs and benefits of such management decisions as climate adaptation and species conservation strategies [89]. Some authors have suggested that assisted colonization and stocking, when applied cautiously and judiciously, could be an essential tool for species conservation in a changing climate [90–92]. Others argued that ecologists do not have the ability to determine when such programs will be successful and whether translocated or rearing individuals will have negative or positive effects on the recipient ecosystems [93, 94]. Regarding main potential drawbacks, it has been shown that straying rates of hatchery fish are higher compared to wild fish, as imprinting may not have been as effective and assisted recolonization can thus increase stray rates of wild populations [95]. As straying represents demographic losses from donor populations, many studies report that large donor hatchery populations are a significant threat to recipient wild populations [96–100]. Concerning allis shad, there is an ongoing stocking program (started in 2008) in the Rhine River (Germany) with juveniles coming from assisted reproduction of wild spawners from the Gironde-Garonne-Dordogne basin (France) [33]; http://www.lanuv.nrw.de/alosa-alosa/en/. Monitoring in the Rhine River reported that spawners have been observed in the river for the first time in more than a half century in 2013 and 2014, while juveniles are commonly caught in the estuary since the beginning of the stocking program [101]. These promising results are in line with the results of the current correlative modelling approach and those of [28] in which the Rhine basin will be suitable for shads around 2100 under climate change scenarios. However, results from the mechanistic SDM suggest that the Rhine basin could be ‘reachable’ by the species from southern populations in timing compatible with the most pessimistic rate of climate change as predicted by RCP 8.5. Applying the precautionary principle, these results would have suggested conservation efforts acting for the recovery of existing populations before trying to rebuild extirpated populations in catchments that could be recolonized naturally by the species such as the Rhine River. Nonetheless, GR3D is a complex model necessarily accompanied by substantial sources of uncertainty (e.g., uncertainties in model parameterization or in modelling choices) and biases (e.g., the GR3D model does not take either evolutionary processes or anthropogenic pressures into account). As such, the outputs of the Rhine stocking program are providing useful and powerful data to improve allis shad knowledge and indirectly the parameterization of the GR3D model. Moreover, considering the poor conservation status of allis shad across its range, we argue that the stocking program in the Rhine basin is a relevant way to improve the species status but that it should be sustained by significant management decisions in neighboring source systems as identified by GR3D.

The mechanistic SDM also showed difficulties in reproducing the species absence in some watersheds. Nevertheless, the correlative model also failed in reproducing species absences in the environment reduced to 73 river basins (i.e., those included in the GR3D physical environment). Most of the watersheds where the species was historically absent were surrounded by catchments with historically observed species presences. Difficulties for both SDMs in reproducing species absences in those basins could be due to imperfect data used during calibration, especially false absence data that require further investigations. As those basins are relatively small, it is possible that allis shad were not surveyed or harvested around 1900. An ecological hypothesis could also be advanced in which finer scale processes, not included in the mechanistic SDM and not linked to one of the predictor variables selected by the correlative SDM, could be involved in local extinction phenomena (e.g., a more successful competitor or predator in a basin with particular environmental characteristics could lead to increase mortality). Local extinction phenomena were demonstrated to play a critical role in species maintenance and it has been demonstrated that increases in river flow amplitude or in river temperature could act on the risk of local extinction for Atlantic salmon *Salmo salar* [102].

In conclusions, our study reveals that, when available, predictions from correlative and mechanistic modelling approaches should be used in a complementary way instead of being opposed. We showed how a combined used of correlative and mechanistic SDMs helps in guiding conservation measures in the climate change context and in identifying data gaps and orienting efforts in data collection (here, on population local adaptation and local extinction phenomena in clupeids). Nonetheless, species for which a mechanistic model with an explicit dispersal process was built and for which potential range shift was studied by combining SDMs outputs remain rare examples. This constitutes a complex, costly and time-consuming work flow that could not be routinely applied. Scientists are still lacking more operational tools to assess whether species will track future climate change appropriately. In this sense, various studies have demonstrated that species’ traits can be important predictors of response to climate change for different taxonomic groups [103–105]. In addition, large numerical databases on species traits have been constructed and were made available online (e.g., FishBase [106]; FishTraits [107]). Building on this strong background, we are currently developing a generic trait-based method to complement more elaborated modelling approaches such as the one presented here in predicting climate change effects on species assemblages.

## Supporting Information

S1 Table Extraction from the EuroDiad 3.2 database for allis shad. ‘Presences_absences’ corresponded to the species historical distribution, ‘Longitude’ and ‘Latitude’ provided the geographic coordinates (°) of the basin outlet, and ‘Surface_area’ was the surface area of the drainage basin in km^2^. In blue cells were given the 73 basins retained in the GR3D physical environment.
(XLSX)

S1 Appendix Calibration of the GR3D model–Full technical details and outcomes of the global sensitivity analysis and optimization steps. (DOCX)
