# Supplementary material for: The Combined Use of Correlative and Mechanistic Species Distribution Models Benefits Low Conservation Status Species
Source: PLoS One. 2015 Oct 1;10(10):e0139194. doi: 10.1371/journal.pone.0139194 (PMC4591278; doi:10.1371/journal.pone.0139194)
Supplement: S1 Appendix — (DOCX) [file pone.0139194.s002.docx]

S1 Appendix: Calibration of the GR3D model – Full technical details and outcomes of the global sensitivity analysis and optimization steps.

For the global sensitivity analysis, maximum and minimum values for the 11 uncertain parameters were determined with the main prerequisite to not exceed the 20% variation commonly used in sensitivity analysis [[1](#_ENREF_1), [2](#_ENREF_2)] (Table 1). A complete sampling with 10 replicates was used meaning that all combinations of uncertain parameters’ values were run and replicated 10 times (i.e. 2^11^ x 10 simulations). During the whole calibration phase, each simulation started with the 73 basins of the GR3D physical environment characterized by mean seasonal water temperatures for the period 1900-1910 and a surface area of the drainage basin. Basins were all populated by 500 000 juveniles at the first time step which was in summer. Simulations lasted 100 years with constant climatic (i.e. temperature) conditions. A variance decomposition-based method was used to conduct the global sensitivity analysis (ANOVA and fisher test to assess parameters’ significance) as these method are regarded as extremely powerful in quantifying the relative importance of input factors and are commonly deployed in ecological modelling studies [[3](#_ENREF_3)]. For each model output response *y*, here the three summary statistics *SS_1_*, *SS_2_* and *SS_3_*, first order indices ($SI$) of an uncertain parameter $x_{i}$ measuring the importance of the variance of the $y$ mean conditionally on factor $x_{i}$, were considered as a sensitivity index [[3](#_ENREF_3), [4](#_ENREF_4)] and were calculated as follows:

. Eq. S1

Global sensitivity index ($TSI$) that measures the mean of the *y* variance on the uncertain parameters different from $x_{i}$ [[3](#_ENREF_3), [4](#_ENREF_4)] were also calculated for each uncertain parameter as follow:

. Eq. S2

TSI corresponds also to a measure of the sum of the main effect of an uncertain parameter $x_{i}$ and the effects of interactions of this parameter with all the other uncertain parameters [[3](#_ENREF_3), [4](#_ENREF_4)]. Both indices $SI$ and $TSI$ were between 0 and 1 but $TSI$ was higher than $SI$ as it included interaction effects. $SI$ and $TSI$ were expressed in percentage and were assumed to express the higher impact on model outputs when they were the more distant from 0. Statistical analysis were carried out using the R software [[5](#_ENREF_5)]. To run all our simulations of the global sensitivity analysis, OpenMOLE 2.0 was used. It is a workflow engine specifically tailored for the distributed exploration of simulation models [[6](#_ENREF_6)].

Based on the two sensitivity indices $SI$ and $TSI$, the 11 uncertain GR3D parameters (Table 1) were classified by decreasing order of variations they caused on the three summary statistics. Two parameters were selected to be calibrated through the optimization step. The selected parameters must have high TSI values on summary statistics and a low interaction between them. Following these considerations, $T_{minRep}$ with a high main effect on *SS1* and *SS2* (*SI* above 20%) and $k_{optGrow}$ explaining more than 80% of the variance for SS3 (with high interactions with $Z_{sea}$ and $L_{mat}$) were selected (Fig A).


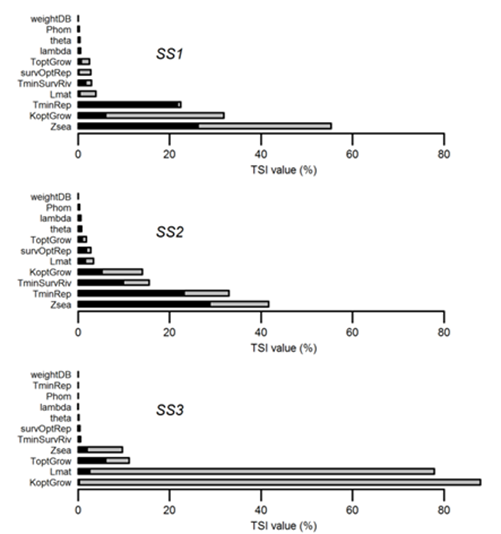


Fig A. Global sensitivity index (TSI) for the 11 uncertain GR3D parameters (in %; the higher the percentage is, the more sensitive the model is to the considered parameter). In black was given the main effect of an uncertain parameter on a summary statistic and in light grey the effect of the same parameter on the same statistic but when considering interactions with other uncertain parameters. Parameters’ abbreviations were presented in Table 1 with theta, lambda and weightDB corresponding to the parameter $\theta$, $\lambda$ and $w^{deathBasin}$ respectively.

For the optimization step, the nine uncertain parameters with the least influence were fixed to the central value of the min-max interval tested during the global sensitivity analysis (Table 1). Then, a uniform distribution between the interval limits was assumed for *T_minrep_* and *k_optGrow_* and constituted the prior for the Bayesian analysis. Indeed, the optimization procedure relied on a recent Approximate Bayesian Computation (ABC) algorithm specifically adapted to complex models and particularly relevant for calibrating stochastic models [[7](#_ENREF_7)]. Approximate Bayesian Computation approximates the likelihood, which is the probability that the data would be obtained from the stochastic model, by the probability that a result close to the data would be obtained from the model. It relied on a large number of repetitions of the three following steps [[8](#_ENREF_8)]: (1) a set of the two influential parameters *T_minrep_* and *k_optGrow_* was randomly drawn according to the parameter prior distributions and a Latin Hypercube Sampling method; (2) simulations of the GR3D model was launched with this parameter set; (3) a set of the three summary statistics *SS1*, *SS2* and *SS3* was computed from these simulations. An acceptance criterion was also defined by the user to select a limited number of simulations which are the closest to data in the space of summary statistics. Once a large number of repetitions of these three steps have been performed, best-fit simulations were defined as the ones that were closest to data in the space of summary statistics. Here, the objective was to search for the sets of influential parameters within the min-max interval that maximized *SS_1_* and that minimized *SS_3_* with both a target value of 0, and that permitted *SS_2_* to get closer to its target value of 53.55°N. The *SS_2_* target value corresponded to the latitude at the outlet of the northern basin where the species was historically recorded present in EuroDiad 3.2, i.e. the Weser River in Germany. The parameter values which have led to these best-fit simulations were finally used to approximate the posterior distributions of the parameters. The new ABC sequential algorithm from Lenormand *et al.* [[7](#_ENREF_7)] implemented in the R package “EasyABC” [[9](#_ENREF_9)] was used to perform this optimization step. The initial number of simulation was set to 4 000 with 3 000 new simulations at each step and a stopping criterion set to 0.01. Algorithm converged after 35 steps (i.e. 106 000 simulations). Posteriors for the two selected parameters were unimodal curves with an optimum at 0.38 cm/season for *k_optGrow_* and 9.8°C for *T_minRep_* (Fig B).


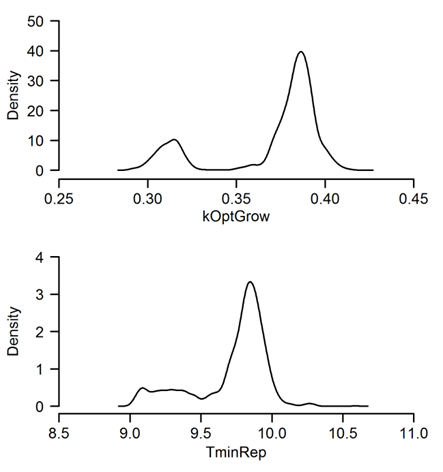


Fig B. Posterior distributions of the two selected influential parameters, *k_optGrow_* and *T_minRep_*.

References

1. Drouineau H, Mahévas S, Pelletier D, Beliaeff B (2006) Assessing the impact of different management options using ISIS-Fish: The French *Merluccius merluccius* - *Nephrops norvegicus* mixed fishery of the Bay of Biscay. Aquat Living Resour 19: 15-29.

2. Lehuta S, Mahévas S, Petitgas P, Pelletier D (2010) Combining sensitivity and uncertainty analysis to evaluate the impact of management measures with ISIS–Fish: Marine protected areas for the Bay of Biscay anchovy (*Engraulis encrasicolus*) fishery. ICES J Mar Sci 67: 1063-1075.

3. Faivre R, Iooss B, Mahévas S, Makowski D, Monod H (2013) Analyse de sensibilité et exploration de modèles - Application aux sciences de la nature et de l'environnement. Versailles: Editions Quae. 352 p.

4. Saltelli A, Ratto M, Andres T, Campolongo F, Cariboni J, Gatelli D, et al. (2008) Global sensitivity analysis: The primer. New York: Wiley. 304 p.

5. R Core Team (2014) R: A language and environment for statistical computing. R Foundation for statistical Computing, Vienna, Austria. URL <http://www.R-project.org/>.

6. Reuillon R, Leclaire M, Rey-Coyrehourcq S (2013) OpenMOLE, a workflow engine specifically tailored for the distributed exploration of simulation models. Future Generation Computer Systems 29: 1981-1990.

7. Lenormand M, Jabot F, Deffuant G (2013) Adaptive approximate Bayesian computation for complex models. Comput Stat 28: 2777-2796.

8. Beaumont MA, Zhang WY, Balding DJ (2002) Approximate Bayesian computation in population genetics. Genetics 162: 2025-2035.

9. Jabot F, Faure T, Dumoulin N (2013) EasyABC: Performing efficient approximate Bayesian computation sampling schemes using R. Methods Ecol Evol 4: 684-687.
